# 2,2-Dimethyl-*N*-(2-methyl­phenyl­sulfon­yl)acetamide

**DOI:** 10.1107/S1600536811027838

**Published:** 2011-07-16

**Authors:** K. Shakuntala, Sabine Foro, B. Thimme Gowda

**Affiliations:** a Department of Chemistry, Mangalore University, Mangalagangotri 574 199, Mangalore, India; b Institute of Materials Science, Darmstadt University of Technology, Petersenstrasse 23, D-64287 Darmstadt, Germany

## Abstract

The asymmetric unit of the title compound, C_11_H_15_NO_3_S, contains two independent mol­ecules in which the amide bonds show a *trans* conformation. The C—S—N—C torsion angles are −67.4 (2) and 63.8 (2)° in the two independent mol­ecules. In one of the mol­ecules, a methyl group is disordered over two sets of sites with a site-occupation factor of 0.661 (16) for the major occupany component. In the crystal, mol­ecules are packed into chains running along [101] through N—H⋯O(S) hydrogen bonds.

## Related literature

For hydrogen bonding modes of sulfonamides, see: Adsmond & Grant (2001 ▶). For our studies on the effects of substituents on the structures of *N*-(ar­yl)-amides, see: Bhat & Gowda (2000 ▶); Gowda *et al.* (2007 ▶), on *N*-(ar­yl)-sulfonamides, see: Gowda *et al.* (2005 ▶), and on *N*-(aryl­sulfon­yl)-amides, see: Shakuntala *et al.* (2011**a* ▶,b* ▶)
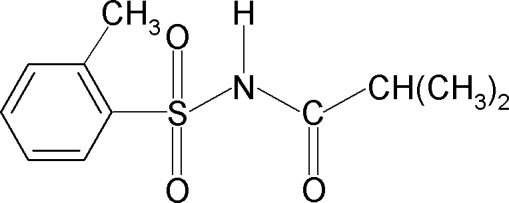


## Experimental

### 

#### Crystal data

C_11_H_15_NO_3_S
*M* *_r_* = 241.30
Monoclinic, 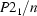

*a* = 11.829 (1) Å
*b* = 16.351 (1) Å
*c* = 13.351 (1) Å
β = 96.485 (8)°
*V* = 2565.8 (3) Å^3^
*Z* = 8
Mo *K*α radiation
μ = 0.25 mm^−1^
*T* = 293 K
0.46 × 0.44 × 0.40 mm

#### Data collection

Oxford Diffraction Xcalibur diffractometer with Sapphire CCD detector
Absorption correction: multi-scan (*CrysAlis RED*; Oxford Diffraction, 2009 ▶) *T*_min_ = 0.896, *T*_max_ = 0.908
9793 measured reflections
5225 independent reflections
3538 reflections with *I* > 2σ(*I*)
*R*_int_ = 0.015

#### Refinement

*R*[*F* ^2^ > 2σ(*F* ^2^)] = 0.045
*wR*(*F* ^2^) = 0.127
*S* = 1.06
5225 reflections
306 parameters
5 restraints
H atoms treated by a mixture of independent and constrained refinement
Δρ_max_ = 0.25 e Å^−3^
Δρ_min_ = −0.32 e Å^−3^

### 

Data collection: *CrysAlis CCD* (Oxford Diffraction, 2009 ▶); cell refinement: *CrysAlis RED* (Oxford Diffraction, 2009 ▶); data reduction: *CrysAlis RED*; program(s) used to solve structure: *SHELXS97* (Sheldrick, 2008 ▶); program(s) used to refine structure: *SHELXL97* (Sheldrick, 2008 ▶); molecular graphics: *PLATON* (Spek, 2009 ▶); software used to prepare material for publication: *SHELXL97*.

## Supplementary Material

Crystal structure: contains datablock(s) I, global. DOI: 10.1107/S1600536811027838/bt5569sup1.cif

Structure factors: contains datablock(s) I. DOI: 10.1107/S1600536811027838/bt5569Isup2.hkl

Supplementary material file. DOI: 10.1107/S1600536811027838/bt5569Isup3.cml

Additional supplementary materials:  crystallographic information; 3D view; checkCIF report

## Figures and Tables

**Table 1:** Hydrogen-bond geometry (Å, °)

*D*—H⋯*A*	*D*—H	H⋯*A*	*D*⋯*A*	*D*—H⋯*A*
N1—H1*N*⋯O6	0.82 (2)	2.02 (2)	2.844 (2)	175 (2)
N2—H2*N*⋯O3^i^	0.81 (2)	2.08 (2)	2.870 (2)	167 (2)

Symmetry code: (i) 
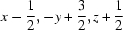
.

**Fig. 1.**
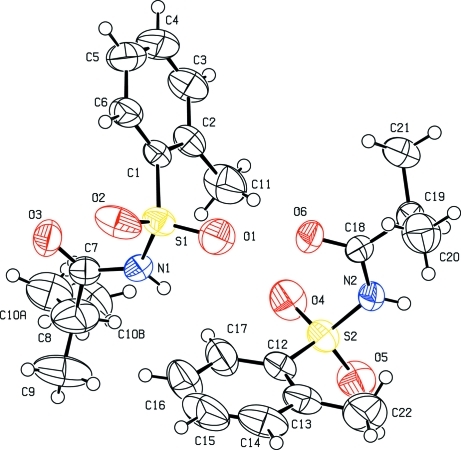
Molecular structure of the title compound, showing the atom- labelling scheme. Displacement ellipsoids are drawn at the 50% probability level.

**Fig. 2.**
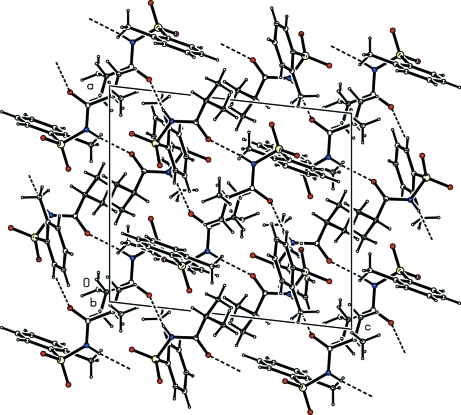
Molecular packing in the title compound. Hydrogen bonds are shown as dashed lines.

## supplementary crystallographic information

### Comment

The hydrogen bonding preferences of sulfonamides has been investigated (Adsmond & Grant, 2001). The nature and position of substituents play a significant role on the crystal structures and other aspects of *N*-(aryl)-amides (Bhat & Gowda, 2000; Gowda *et al.*, 2007), *N*-(aryl)-sulfonamides (Gowda *et al.*, 2005) and *N*-(arylsulfonyl)-amides (Shakuntala *et al.*, 2011**a*,b*). As a part of studying the effects of substituents on the structures of this class of compounds, the structure of *N*-(2-methylphenylsulfonyl)-2,2-dimethylacetamide (I) has been determined (Fig. 1). In (I), the asymmetric unit contains two independent molecules. The conformations of the N—H and C=O bonds in the side chains are anti to each other, similar to that observed in *N*-(2-methylphenylsulfonyl)-acetamide (II) (Shakuntala *et al.*, 2011*b*) and *N*-(2-chlorophenylsulfonyl)-2,2-dimethylacetamide (III) (Shakuntala *et al.*, 2011*a*).

Further, in both the independent molecules, the conformation of the amide H atoms are *syn* to the *ortho*-methyl groups in the benzene rings, similar to that observed between the amide H atom and the *ortho*-methyl group in (II), but contrary to the *anti* conformation observed between the amide H atom and the *ortho*-chloro group in (III).

The molecules in (I) are bent at the *S*-atoms with a C—S—N—C torsion angle of -67.4 (2)° and 63.8 (2)° in the two independent molecules, compared to the values of -58.2 (2)° in (II) and 64.4 (2)° in (III). Further, the dihedral angle between the benzene rings and the SO2—NH—CO—C groups in (I) are 86.1 (2)° (molecule 1) and 87.4 (2)° (molecule 2), compared to the values of 87.0 (1)° in (II) and 87.4 (1)° in (III).

In the crystal structure, the intermolecular N–H···O hydrogen bonds (Table 1) link the molecules into chains. Part of the crystal structure is shown in Fig. 2.

### Experimental

The title compound was prepared by refluxing 2-methylbenzenesulfonamide (0.10 mole) with an excess of 2,2-dimethylacetyl chloride (0.20 mole) for one hour on a water bath. The reaction mixture was cooled and poured into ice cold water. The resulting solid was separated, washed thoroughly with water and dissolved in warm dilute sodium hydrogen carbonate solution. The title compound was reprecipitated by acidifying the filtered solution with glacial acetic acid. It was filtered, dried and recrystallized from ethanol. The purity of the compound was checked by determining its melting point. It was further characterized by recording its infrared spectra.

Prism like colorless single crystals of the title compound used in X-ray diffraction studies were obtained from a slow evaporation of an ethanolic solution of the compound.

### Refinement

The H atoms of the NH groups were located in a difference map and later restrained to the distance N—H = 0.86 (2) Å. The other H atoms were positioned with idealized geometry using a riding model the with aromatic C—H distance = 0.93 Å, methyl C—H = 0.96 Å, methyne C—H = 0.98 Å. All H atoms were refined with isotropic displacement parameters (set to 1.2 times of the *U*_eq_ of the parent atom).

The atom C10 is disordered and was refined using a split model. The corresponding site-occupation factors were refined so that their sum was unity [0.66 (2) and 0.34 (2)]. The distance C8—C10 was restrained to 1.55 (1) Å and the corresponding bond distances in the disordered group were restrained to be equal.

### Crystal data

**Table:**

C_11_H_15_NO_3_S	*F*(000) = 1024
*M**_r_* = 241.30	*D*_x_ = 1.249 Mg m^−^^3^
Monoclinic, *P*2_1_/*n*	Mo *K*α radiation, λ = 0.71073 Å
Hall symbol: -P 2yn	Cell parameters from 2934 reflections
*a* = 11.829 (1) Å	θ = 2.9–27.7°
*b* = 16.351 (1) Å	µ = 0.25 mm^−^^1^
*c* = 13.351 (1) Å	*T* = 293 K
β = 96.485 (8)°	Prism, colourless
*V* = 2565.8 (3) Å^3^	0.46 × 0.44 × 0.40 mm
*Z* = 8	

### Data collection

**Table:**

Oxford Diffraction Xcalibur diffractometer with Sapphire CCD detector	5225 independent reflections
Radiation source: fine-focus sealed tube	3538 reflections with *I* > 2σ(*I*)
graphite	*R*_int_ = 0.015
Rotation method data acquisition using ω and φ scans	θ_max_ = 26.4°, θ_min_ = 2.9°
Absorption correction: multi-scan (*CrysAlis RED*; Oxford Diffraction, 2009)	*h* = −14→14
*T*_min_ = 0.896, *T*_max_ = 0.908	*k* = −20→15
9793 measured reflections	*l* = −16→14

### Refinement

**Table:**

Refinement on *F*^2^	Secondary atom site location: difference Fourier map
Least-squares matrix: full	Hydrogen site location: inferred from neighbouring sites
*R*[*F*^2^ > 2σ(*F*^2^)] = 0.045	H atoms treated by a mixture of independent and constrained refinement
*wR*(*F*^2^) = 0.127	*w* = 1/[σ^2^(*F*_o_^2^) + (0.0663*P*)^2^ + 0.310*P*] where *P* = (*F*_o_^2^ + 2*F*_c_^2^)/3
*S* = 1.06	(Δ/σ)_max_ = 0.001
5225 reflections	Δρ_max_ = 0.25 e Å^−^^3^
306 parameters	Δρ_min_ = −0.32 e Å^−^^3^
5 restraints	Extinction correction: *SHELXL97* (Sheldrick, 2008), Fc^*^=kFc[1+0.001xFc^2^λ^3^/sin(2θ)]^-1/4^
Primary atom site location: structure-invariant direct methods	Extinction coefficient: 0.0160 (11)

### Special details

**Table:**

Geometry. All e.s.d.'s (except the e.s.d. in the dihedral angle between two l.s. planes) are estimated using the full covariance matrix. The cell e.s.d.'s are taken into account individually in the estimation of e.s.d.'s in distances, angles and torsion angles; correlations between e.s.d.'s in cell parameters are only used when they are defined by crystal symmetry. An approximate (isotropic) treatment of cell e.s.d.'s is used for estimating e.s.d.'s involving l.s. planes.
Refinement. Refinement of *F*^2^ against ALL reflections. The weighted *R*-factor *wR* and goodness of fit *S* are based on *F*^2^, conventional *R*-factors *R* are based on *F*, with *F* set to zero for negative *F*^2^. The threshold expression of *F*^2^ > σ(*F*^2^) is used only for calculating *R*-factors(gt) *etc*. and is not relevant to the choice of reflections for refinement. *R*-factors based on *F*^2^ are statistically about twice as large as those based on *F*, and *R*- factors based on ALL data will be even larger.

### Fractional atomic coordinates and isotropic or equivalent isotropic displacement parameters (Å^2^)

**Table:**

	*x*	*y*	*z*	*U*_iso_*/*U*_eq_	Occ. (<1)
C1	0.24942 (16)	0.90807 (11)	0.25752 (15)	0.0439 (5)	
C2	0.24885 (19)	0.97836 (13)	0.31734 (18)	0.0566 (6)	
C3	0.2774 (2)	1.05064 (15)	0.2737 (2)	0.0771 (8)	
H3	0.2784	1.0986	0.3112	0.093*	
C4	0.3040 (3)	1.05412 (18)	0.1779 (3)	0.0960 (10)	
H4	0.3225	1.1042	0.1512	0.115*	
C5	0.3042 (3)	0.98543 (19)	0.1197 (2)	0.0959 (10)	
H5	0.3230	0.9886	0.0540	0.115*	
C6	0.2762 (2)	0.91095 (15)	0.16000 (18)	0.0641 (6)	
H6	0.2755	0.8635	0.1215	0.077*	
C7	0.3989 (2)	0.77526 (15)	0.41066 (16)	0.0557 (6)	
C8	0.4565 (3)	0.7573 (2)	0.5134 (2)	0.0990 (10)	
H8A	0.4036	0.7839	0.5547	0.119*	0.661 (16)
H8B	0.4179	0.7894	0.5617	0.119*	0.339 (16)
C9	0.4490 (4)	0.6760 (2)	0.5464 (3)	0.1276 (15)	
H9A	0.4842	0.6403	0.5018	0.153*	
H9B	0.3705	0.6611	0.5464	0.153*	
H9C	0.4873	0.6709	0.6134	0.153*	
C10A	0.5512 (10)	0.8134 (5)	0.5426 (7)	0.121 (4)	0.661 (16)
H10A	0.5244	0.8688	0.5369	0.145*	0.661 (16)
H10B	0.6094	0.8054	0.4989	0.145*	0.661 (16)
H10C	0.5818	0.8026	0.6110	0.145*	0.661 (16)
C10B	0.4852 (11)	0.8264 (5)	0.5782 (8)	0.072 (3)	0.339 (16)
H10D	0.5199	0.8075	0.6425	0.086*	0.339 (16)
H10E	0.4174	0.8566	0.5871	0.086*	0.339 (16)
H10F	0.5375	0.8612	0.5482	0.086*	0.339 (16)
C11	0.2176 (3)	0.97897 (18)	0.4234 (2)	0.0941 (10)	
H11A	0.2694	0.9447	0.4651	0.113*	
H11B	0.1415	0.9587	0.4238	0.113*	
H11C	0.2220	1.0339	0.4491	0.113*	
N1	0.28574 (16)	0.79150 (11)	0.40442 (13)	0.0498 (4)	
H1N	0.2555 (19)	0.7959 (15)	0.4568 (14)	0.060*	
O1	0.09374 (14)	0.82144 (12)	0.32966 (16)	0.0884 (6)	
O2	0.22413 (17)	0.75295 (9)	0.22545 (13)	0.0776 (5)	
O3	0.44952 (15)	0.77645 (13)	0.33654 (13)	0.0803 (6)	
S1	0.20407 (5)	0.81269 (3)	0.29885 (4)	0.05239 (18)	
C12	0.25372 (19)	0.94032 (13)	0.75834 (16)	0.0540 (5)	
C13	0.1651 (2)	0.99676 (15)	0.7539 (2)	0.0689 (7)	
C14	0.1867 (3)	1.07325 (18)	0.7135 (2)	0.0941 (10)	
H14	0.1293	1.1124	0.7074	0.113*	
C15	0.2888 (4)	1.0921 (2)	0.6828 (3)	0.1094 (12)	
H15	0.2998	1.1437	0.6563	0.131*	
C16	0.3756 (3)	1.0370 (2)	0.6901 (3)	0.1032 (11)	
H16	0.4458	1.0510	0.6700	0.124*	
C17	0.3575 (2)	0.96009 (17)	0.7278 (2)	0.0770 (8)	
H17	0.4157	0.9215	0.7326	0.092*	
C18	0.12222 (18)	0.78145 (13)	0.64273 (16)	0.0475 (5)	
C19	0.0175 (2)	0.73490 (15)	0.60130 (17)	0.0587 (6)	
H19	−0.0153	0.7092	0.6577	0.070*	
C20	−0.0680 (2)	0.7950 (2)	0.5511 (3)	0.0923 (9)	
H20A	−0.0360	0.8223	0.4972	0.111*	
H20B	−0.0867	0.8346	0.5997	0.111*	
H20C	−0.1356	0.7663	0.5246	0.111*	
C21	0.0469 (3)	0.66856 (17)	0.5299 (2)	0.0847 (9)	
H21A	0.1004	0.6314	0.5650	0.102*	
H21B	0.0799	0.6927	0.4745	0.102*	
H21C	−0.0209	0.6394	0.5049	0.102*	
C22	0.0524 (2)	0.9796 (2)	0.7900 (3)	0.1058 (11)	
H22A	0.0632	0.9669	0.8606	0.127*	
H22B	0.0172	0.9341	0.7534	0.127*	
H22C	0.0045	1.0269	0.7791	0.127*	
N2	0.13051 (15)	0.79850 (11)	0.74326 (13)	0.0484 (4)	
H2N	0.0846 (17)	0.7801 (14)	0.7778 (16)	0.058*	
O4	0.34155 (14)	0.79679 (11)	0.79255 (15)	0.0763 (5)	
O5	0.21005 (16)	0.84663 (12)	0.90834 (12)	0.0793 (5)	
O6	0.19510 (14)	0.80550 (11)	0.59210 (12)	0.0650 (5)	
S2	0.24242 (5)	0.84139 (4)	0.80908 (4)	0.05448 (19)	

### Atomic displacement parameters (Å^2^)

**Table:**

	*U*^11^	*U*^22^	*U*^33^	*U*^12^	*U*^13^	*U*^23^
C1	0.0460 (11)	0.0338 (10)	0.0508 (12)	0.0038 (9)	0.0004 (9)	0.0021 (8)
C2	0.0646 (14)	0.0393 (12)	0.0641 (14)	0.0062 (10)	−0.0001 (11)	−0.0043 (10)
C3	0.099 (2)	0.0351 (13)	0.097 (2)	−0.0028 (13)	0.0053 (16)	−0.0023 (13)
C4	0.123 (3)	0.0485 (17)	0.121 (3)	−0.0030 (17)	0.034 (2)	0.0257 (17)
C5	0.136 (3)	0.075 (2)	0.085 (2)	0.0200 (19)	0.050 (2)	0.0288 (17)
C6	0.0875 (18)	0.0487 (14)	0.0581 (14)	0.0144 (12)	0.0162 (12)	0.0046 (11)
C7	0.0590 (14)	0.0630 (15)	0.0451 (12)	0.0084 (11)	0.0056 (10)	0.0098 (10)
C8	0.108 (2)	0.123 (3)	0.0601 (18)	−0.004 (2)	−0.0171 (16)	0.0297 (18)
C9	0.168 (4)	0.113 (3)	0.092 (2)	−0.006 (3)	−0.028 (2)	0.054 (2)
C10A	0.142 (8)	0.126 (5)	0.081 (4)	−0.010 (5)	−0.043 (5)	0.018 (4)
C10B	0.058 (6)	0.089 (7)	0.066 (6)	−0.001 (5)	0.000 (4)	−0.005 (4)
C11	0.150 (3)	0.0631 (18)	0.0695 (18)	0.0237 (18)	0.0144 (18)	−0.0181 (13)
N1	0.0553 (11)	0.0534 (11)	0.0426 (10)	0.0039 (8)	0.0140 (8)	0.0064 (8)
O1	0.0473 (10)	0.0915 (15)	0.1265 (17)	−0.0073 (9)	0.0104 (10)	0.0322 (12)
O2	0.1242 (15)	0.0351 (8)	0.0670 (11)	−0.0057 (9)	−0.0176 (10)	−0.0091 (7)
O3	0.0660 (11)	0.1159 (16)	0.0623 (11)	0.0321 (10)	0.0218 (9)	0.0169 (10)
S1	0.0526 (3)	0.0400 (3)	0.0622 (4)	−0.0057 (2)	−0.0041 (2)	0.0048 (2)
C12	0.0569 (13)	0.0462 (12)	0.0566 (13)	−0.0050 (10)	−0.0041 (10)	−0.0071 (10)
C13	0.0740 (17)	0.0484 (14)	0.0807 (18)	0.0047 (12)	−0.0070 (13)	−0.0155 (12)
C14	0.126 (3)	0.0484 (17)	0.101 (2)	0.0135 (18)	−0.016 (2)	−0.0136 (15)
C15	0.165 (4)	0.0490 (18)	0.113 (3)	−0.026 (2)	0.009 (3)	−0.0052 (17)
C16	0.112 (3)	0.077 (2)	0.123 (3)	−0.042 (2)	0.022 (2)	−0.0035 (19)
C17	0.0714 (17)	0.0647 (17)	0.095 (2)	−0.0182 (14)	0.0082 (14)	−0.0027 (14)
C18	0.0501 (12)	0.0457 (12)	0.0485 (12)	−0.0006 (9)	0.0134 (10)	−0.0045 (9)
C19	0.0616 (14)	0.0678 (16)	0.0490 (13)	−0.0193 (12)	0.0164 (11)	−0.0106 (11)
C20	0.0634 (17)	0.106 (2)	0.104 (2)	−0.0036 (17)	−0.0055 (15)	−0.0181 (19)
C21	0.102 (2)	0.0694 (19)	0.084 (2)	−0.0232 (16)	0.0195 (16)	−0.0267 (14)
C22	0.075 (2)	0.087 (2)	0.154 (3)	0.0248 (17)	0.009 (2)	−0.029 (2)
N2	0.0493 (11)	0.0518 (11)	0.0455 (10)	−0.0092 (8)	0.0117 (8)	−0.0011 (8)
O4	0.0547 (10)	0.0647 (11)	0.1057 (14)	0.0137 (8)	−0.0069 (9)	0.0100 (10)
O5	0.0944 (14)	0.0959 (14)	0.0447 (9)	−0.0164 (11)	−0.0041 (8)	0.0007 (9)
O6	0.0611 (10)	0.0820 (12)	0.0556 (9)	−0.0186 (9)	0.0232 (8)	−0.0101 (8)
S2	0.0530 (3)	0.0532 (3)	0.0549 (3)	−0.0023 (3)	−0.0044 (2)	0.0031 (2)

### Geometric parameters (Å, °)

**Table:**

C1—C6	1.375 (3)	N1—H1N	0.824 (16)
C1—C2	1.400 (3)	O1—S1	1.4193 (18)
C1—S1	1.758 (2)	O2—S1	1.4224 (17)
C2—C3	1.377 (3)	C12—C17	1.375 (3)
C2—C11	1.504 (3)	C12—C13	1.393 (3)
C3—C4	1.352 (4)	C12—S2	1.765 (2)
C3—H3	0.9300	C13—C14	1.397 (4)
C4—C5	1.366 (4)	C13—C22	1.493 (4)
C4—H4	0.9300	C14—C15	1.354 (5)
C5—C6	1.387 (4)	C14—H14	0.9300
C5—H5	0.9300	C15—C16	1.361 (5)
C6—H6	0.9300	C15—H15	0.9300
C7—O3	1.214 (2)	C16—C17	1.381 (4)
C7—N1	1.357 (3)	C16—H16	0.9300
C7—C8	1.491 (3)	C17—H17	0.9300
C8—C9	1.407 (5)	C18—O6	1.219 (2)
C8—C10B	1.439 (7)	C18—N2	1.363 (3)
C8—C10A	1.465 (6)	C18—C19	1.505 (3)
C8—H8A	0.9800	C19—C21	1.510 (3)
C8—H8B	0.9815	C19—C20	1.512 (4)
C9—H9A	0.9600	C19—H19	0.9800
C9—H9B	0.9600	C20—H20A	0.9600
C9—H9C	0.9600	C20—H20B	0.9600
C10A—H10A	0.9600	C20—H20C	0.9600
C10A—H10B	0.9600	C21—H21A	0.9600
C10A—H10C	0.9600	C21—H21B	0.9600
C10B—H8A	1.2021	C21—H21C	0.9600
C10B—H8B	1.0050	C22—H22A	0.9600
C10B—H10D	0.9600	C22—H22B	0.9600
C10B—H10E	0.9600	C22—H22C	0.9600
C10B—H10F	0.9600	N2—S2	1.6594 (18)
C11—H11A	0.9600	N2—H2N	0.809 (15)
C11—H11B	0.9600	O4—S2	1.4191 (17)
C11—H11C	0.9600	O5—S2	1.4230 (18)
N1—S1	1.6530 (19)		
			
C6—C1—C2	121.9 (2)	C2—C11—H11C	109.5
C6—C1—S1	116.01 (16)	H11A—C11—H11C	109.5
C2—C1—S1	121.86 (17)	H11B—C11—H11C	109.5
C3—C2—C1	116.5 (2)	C7—N1—S1	124.78 (15)
C3—C2—C11	119.4 (2)	C7—N1—H1N	119.0 (17)
C1—C2—C11	124.1 (2)	S1—N1—H1N	116.0 (17)
C4—C3—C2	122.1 (3)	O1—S1—O2	119.96 (13)
C4—C3—H3	119.0	O1—S1—N1	104.00 (11)
C2—C3—H3	119.0	O2—S1—N1	108.46 (10)
C3—C4—C5	121.3 (3)	O1—S1—C1	108.94 (11)
C3—C4—H4	119.4	O2—S1—C1	108.20 (11)
C5—C4—H4	119.4	N1—S1—C1	106.49 (9)
C4—C5—C6	119.0 (3)	C17—C12—C13	121.7 (2)
C4—C5—H5	120.5	C17—C12—S2	115.98 (19)
C6—C5—H5	120.5	C13—C12—S2	122.29 (19)
C1—C6—C5	119.2 (2)	C12—C13—C14	116.1 (3)
C1—C6—H6	120.4	C12—C13—C22	123.8 (2)
C5—C6—H6	120.4	C14—C13—C22	120.1 (3)
O3—C7—N1	121.4 (2)	C15—C14—C13	122.0 (3)
O3—C7—C8	122.5 (2)	C15—C14—H14	119.0
N1—C7—C8	116.1 (2)	C13—C14—H14	119.0
C9—C8—C10B	124.9 (5)	C14—C15—C16	121.3 (3)
C9—C8—C10A	125.5 (4)	C14—C15—H15	119.4
C10B—C8—C10A	39.5 (4)	C16—C15—H15	119.4
C9—C8—C7	115.8 (3)	C15—C16—C17	118.9 (3)
C10B—C8—C7	116.8 (5)	C15—C16—H16	120.6
C10A—C8—C7	112.2 (3)	C17—C16—H16	120.6
C9—C8—H8A	100.1	C12—C17—C16	120.2 (3)
C10B—C8—H8A	55.8	C12—C17—H17	119.9
C10A—C8—H8A	95.2	C16—C17—H17	119.9
C7—C8—H8A	100.1	O6—C18—N2	120.2 (2)
C9—C8—H8B	104.4	O6—C18—C19	124.29 (19)
C10B—C8—H8B	44.2	N2—C18—C19	115.47 (17)
C10A—C8—H8B	83.7	C18—C19—C21	110.9 (2)
C7—C8—H8B	107.5	C18—C19—C20	108.4 (2)
H8A—C8—H8B	11.9	C21—C19—C20	112.2 (2)
C8—C9—H9A	109.5	C18—C19—H19	108.4
C8—C9—H9B	109.5	C21—C19—H19	108.4
H9A—C9—H9B	109.5	C20—C19—H19	108.4
C8—C9—H9C	109.5	C19—C20—H20A	109.5
H9A—C9—H9C	109.5	C19—C20—H20B	109.5
H9B—C9—H9C	109.5	H20A—C20—H20B	109.5
C8—C10A—H10A	109.5	C19—C20—H20C	109.5
C8—C10A—H10B	109.5	H20A—C20—H20C	109.5
H10A—C10A—H10B	109.5	H20B—C20—H20C	109.5
C8—C10A—H10C	109.5	C19—C21—H21A	109.5
H10A—C10A—H10C	109.5	C19—C21—H21B	109.5
H10B—C10A—H10C	109.5	H21A—C21—H21B	109.5
C8—C10B—H8A	42.4	C19—C21—H21C	109.5
C8—C10B—H8B	42.9	H21A—C21—H21C	109.5
H8A—C10B—H8B	2.7	H21B—C21—H21C	109.5
C8—C10B—H10D	109.5	C13—C22—H22A	109.5
H8A—C10B—H10D	107.3	C13—C22—H22B	109.5
H8B—C10B—H10D	104.5	H22A—C22—H22B	109.5
C8—C10B—H10E	109.5	C13—C22—H22C	109.5
H8A—C10B—H10E	70.9	H22A—C22—H22C	109.5
H8B—C10B—H10E	71.6	H22B—C22—H22C	109.5
H10D—C10B—H10E	109.5	C18—N2—S2	124.90 (14)
C8—C10B—H10F	109.5	C18—N2—H2N	120.5 (17)
H8A—C10B—H10F	140.3	S2—N2—H2N	113.7 (17)
H8B—C10B—H10F	142.8	O4—S2—O5	119.32 (12)
H10D—C10B—H10F	109.5	O4—S2—N2	108.92 (10)
H10E—C10B—H10F	109.5	O5—S2—N2	103.66 (10)
C2—C11—H11A	109.5	O4—S2—C12	108.15 (11)
C2—C11—H11B	109.5	O5—S2—C12	110.02 (11)
H11A—C11—H11B	109.5	N2—S2—C12	105.94 (10)
			
C6—C1—C2—C3	−0.5 (3)	C2—C1—S1—N1	−58.61 (19)
S1—C1—C2—C3	−174.84 (18)	C17—C12—C13—C14	2.2 (4)
C6—C1—C2—C11	178.5 (2)	S2—C12—C13—C14	178.89 (19)
S1—C1—C2—C11	4.1 (3)	C17—C12—C13—C22	−177.4 (3)
C1—C2—C3—C4	0.4 (4)	S2—C12—C13—C22	−0.7 (3)
C11—C2—C3—C4	−178.7 (3)	C12—C13—C14—C15	−1.7 (4)
C2—C3—C4—C5	−0.3 (5)	C22—C13—C14—C15	177.9 (3)
C3—C4—C5—C6	0.2 (5)	C13—C14—C15—C16	0.0 (5)
C2—C1—C6—C5	0.5 (4)	C14—C15—C16—C17	1.2 (5)
S1—C1—C6—C5	175.1 (2)	C13—C12—C17—C16	−1.1 (4)
C4—C5—C6—C1	−0.3 (5)	S2—C12—C17—C16	−178.0 (2)
O3—C7—C8—C9	−96.8 (4)	C15—C16—C17—C12	−0.7 (5)
N1—C7—C8—C9	84.1 (4)	O6—C18—C19—C21	44.9 (3)
O3—C7—C8—C10B	100.0 (8)	N2—C18—C19—C21	−137.2 (2)
N1—C7—C8—C10B	−79.1 (8)	O6—C18—C19—C20	−78.7 (3)
O3—C7—C8—C10A	56.6 (8)	N2—C18—C19—C20	99.2 (2)
N1—C7—C8—C10A	−122.5 (7)	O6—C18—N2—S2	−7.8 (3)
O3—C7—N1—S1	1.1 (3)	C19—C18—N2—S2	174.15 (16)
C8—C7—N1—S1	−179.8 (2)	C18—N2—S2—O4	−52.3 (2)
C7—N1—S1—O1	177.60 (19)	C18—N2—S2—O5	179.70 (18)
C7—N1—S1—O2	48.9 (2)	C18—N2—S2—C12	63.9 (2)
C7—N1—S1—C1	−67.4 (2)	C17—C12—S2—O4	−9.3 (2)
C6—C1—S1—O1	−121.66 (19)	C13—C12—S2—O4	173.83 (19)
C2—C1—S1—O1	53.0 (2)	C17—C12—S2—O5	122.6 (2)
C6—C1—S1—O2	10.3 (2)	C13—C12—S2—O5	−54.3 (2)
C2—C1—S1—O2	−175.03 (17)	C17—C12—S2—N2	−125.97 (19)
C6—C1—S1—N1	126.72 (17)	C13—C12—S2—N2	57.2 (2)

### Hydrogen-bond geometry (Å, °)

**Table:**

*D*—H···*A*	*D*—H	H···*A*	*D*···*A*	*D*—H···*A*
N1—H1N···O6	0.82 (2)	2.02 (2)	2.844 (2)	175 (2)
N2—H2N···O3^i^	0.81 (2)	2.08 (2)	2.870 (2)	167 (2)

Symmetry codes: (i) *x*−1/2, −*y*+3/2, *z*+1/2.
